# Endothelial Cells as a Key Cell Type for Innate Immunity: A Focused Review on RIG-I Signaling Pathway

**DOI:** 10.3389/fimmu.2022.951614

**Published:** 2022-07-05

**Authors:** Suowen Xu, Tengchuan Jin, Jianping Weng

**Affiliations:** ^1^ Department of Endocrinology, Institute of Endocrine and Metabolic Diseases, The First Affiliated Hospital of USTC, Division of Life Sciences and Medicine, Clinical Research Hospital of Chinese Academy of Sciences (Hefei), University of Science and Technology of China, Hefei, China; ^2^ Laboratory of Metabolics and Cardiovascular Diseases, Institute of Endocrine and Metabolic Diseases, University of Science and Technology of China, Hefei, China; ^3^ Biomedical Sciences and Health Laboratory of Anhui Province , University of Science and Technology of China, Hefei, China; ^4^ Laboratory of Structural Immunology, Division of Life Sciences and Medicine, University of Science and Technology of China, Hefei, China

**Keywords:** RIG-I, DDX58, immunity, endothelial cells, inflammation

## Abstract

The vascular endothelium consists of a highly heterogeneous monolayer of endothelial cells (ECs) which are the primary target for bacterial and viral infections due to EC’s constant and close contact with the bloodstream. Emerging evidence has shown that ECs are a key cell type for innate immunity. Like macrophages, ECs serve as sentinels when sensing invading pathogens or microbial infection caused by viruses and bacteria. It remains elusive how ECs senses danger signals, transduce the signal and fulfil immune functions. Retinoic acid-inducible gene-I (RIG-I, gene name also known as DDX58) is an important member of RIG-I-like receptor (RLR) family that functions as an important pathogen recognition receptor (PRR) to execute immune surveillance and confer host antiviral response. Recent studies have demonstrated that virus infection, dsRNA, dsDNA, interferons, LPS, and 25-hydroxycholesterol (25-HC) can increase RIG-1 expression in ECs and propagate anti-viral response. Of translational significance, RIG-I activation can be inhibited by *Panax notoginseng* saponins, endogenous PPARγ ligand 15-PGJ2, tryptanthrin and 2-animopurine. Considering the pivotal role of inflammation and innate immunity in regulating endothelial dysfunction and atherosclerosis, here we provided a concise review of the role of RIG-I in endothelial cell function and highlight future direction to elucidate the potential role of RIG-I in regulating cardiovascular diseases as well as virus infectious disease, including COVID-19. Furthered understanding of RIG-I-mediated signaling pathways is important to control disorders associated with altered immunity and inflammation in ECs.

## Introduction

Endothelial cells (ECs) are the innermost cell type lining the blood vessel, allowing for its frequent interactions with substances in the flowing blood (including leukocytes, platelets, bacteria and viruses) under healthy and diseased conditions (1). Similar as the epithelium in the lung and intestine, the endothelium serves as a physical barrier between circulating blood components and vascular wall, thereby acting as a gatekeeper of vascular health and function (2). As one of the major cell types in the blood vessel, ECs play vital roles in regulating metabolism, detoxification, antioxidant redox status, inflammation and immunity responses. ECs execute these functions *via* secreting various bioactive molecules, including proteins that regulate vascular tone, bactericidal proteins, and opsonins that assist in the phagocytosis of foreign bacteria/virus/dead cells/cell debris. ECs maintain vascular homeostasis by expressing molecules associated with vascular homeostasis-associated molecular patterns (3, 4). It is imaginable that once exposed to cardiovascular risk factors (hyperlipidemia, hyperglycemia, smoking, sedentary life style, aging etc) and invading pathogens (such as bacteria, SRAS-CoV, SRAS-CoV2, denge virus etc.), the ECs can sense these danger-associated molecular patterns (DAMP) and pathogen-associated molecular patterns (PAMP), thus transduce the innate immunity related signaling pathways to counteract the deleterious effects of these endogenous and exogenous danger molecules. Once this counter measure is defective, the harmful effects will continue to surge and immune disorders perpetuate in multiple tissues and organs (5).

Atherosclerosis, as the pathological basis of cardiovascular diseases, has long been deemed as an inflammatory and immune diseases with the functional and complex interplay among ECs and monocytes, neutrophils and platelets (1). Under normal condition, EC functions are intact, which allow EC to exert its function in vascular homeostasis. However, once activated by inflammatory stimuli and danger signals transduced by cardiovascular risk factors, a plethora of adhesion/chemotactic molecules such as intercellular adhesion molecule-1 (ICAM-1), vascular cell adhesion molecule-1 (VCAM-1), monocyte chemoattractant protein-1 (MCP-1), E-selectin and P-selectin are upregulated, which trigger leukocyte adhesion to inflamed endothelium, rolling on the cell surface, and diapedesis into sub-endothelium space (6). This process of endothelial activation was finely tuned by the effects of various cytokines/chemokines. For example, IL-17A promotes endothelial inflammation and activation *via* p38-MAPK pathway (7). However, IL-35, an anti-inflammatory cytokine, can inhibit LPS and lysophosphatidylcholine (LPC)-induced endothelial inflammation and activation *via* suppressing the activation of MAPK/AP-1 pathway and epigenetic mechanisms (8–10). ECs can facilitate the phagocytosis of LDL and its oxidatively modified form-oxLDL, leading to endothelial dysfunction (11). LDL can also undergo transcytosis across EC membrane by receptors such as SR-BI and ALK (5, 12). During the advanced stage of atherosclerosis, cardiovascular risk factors can promote endothelial apoptosis, leading to endothelial denudation, plaque erosion and thrombus formation (5).

**Figure 1:**
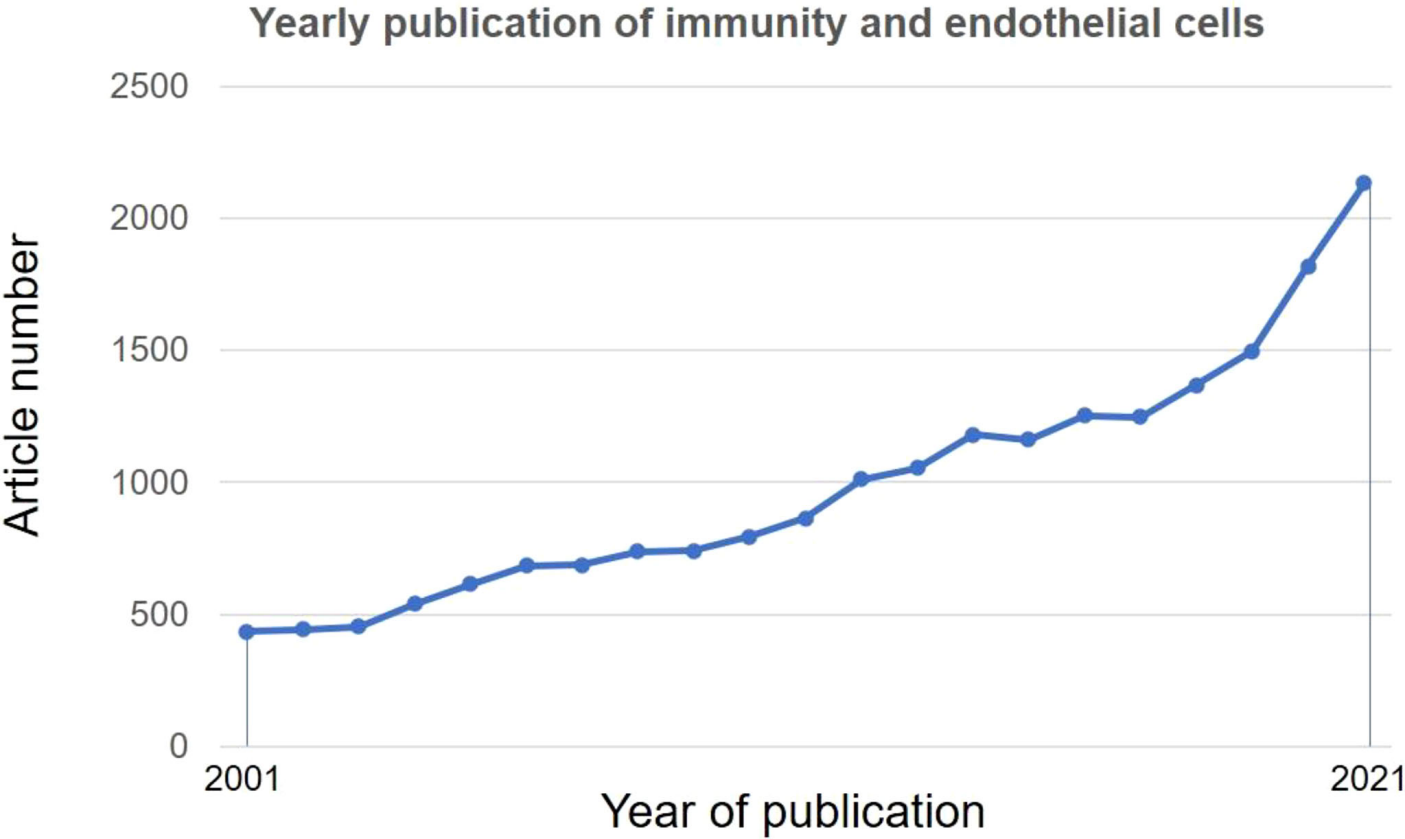
Research trends of immune functions of endothelial cells. A literature search was performed in PubMed using subject terms: “Immune” OR “Immunity” AND “endothelial cell”. Data were retrieved on May 18, 2022.

Given the increased number of publications and appreciation of ECs in regulating immune functions (13) (**Figure 1**) and the position of atherosclerosis as an inflammatory and immune disease (14), molecular underpinnings of immune functions of ECs are pivotal for devising novel immune-target future therapies for atherosclerosis.

## RIG-I Signaling Pathway: A Brief Overview

**Figure 2:**
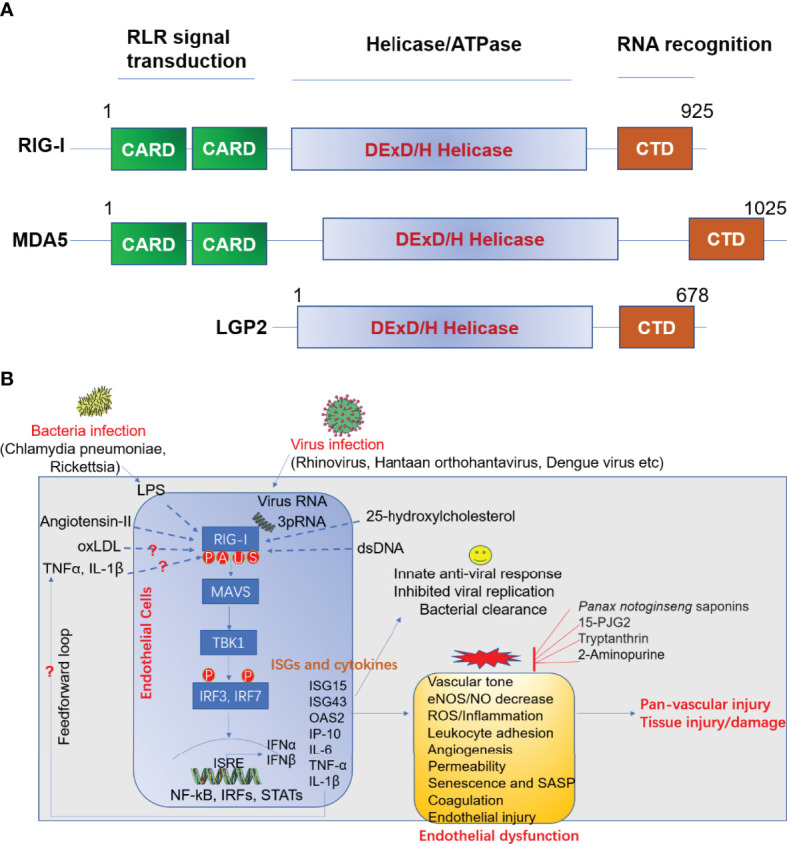
**(A)** Domain structure of RIG-I like receptor (RLR). **(B)** Role of RIG-I in anti-viral response and endothelial dysfunction. LPS, lipopolysaccharide; oxLDL, oxidized LDL; TNFα, tumor necrosis factor α; IL-1β, interleukin 1 beta; RIG-I, retinoic acid-inducible gene I; MAVS, mitochondrial antiviral signaling; TBK1, TANK-binding kinase 1; IRF, interferon response factor; ISRE, interferon-sensitive responsive element; NF-κB, nuclear factor kappa B; STAT, signal transducer and activator of transcription; ISG, interferon-stimulated gene; IL-6, interleukin-6; IP-10 (also known as CXCL10), Interferon gamma-induced protein 10; eNOS, endothelial nitric oxide synthase; NO, nitric oxide; 15-dPGJ2, 15-deoxy-delta-12,14-prostaglandin J2; ROS, reactive oxygen species; SASP, senescence-associated secretory phenotype; IFN, interferon; OAS2, 2’-5’-oligoadenylate synthetase 2; MDA5, melanoma differentiation-associated protein 5; LGP2, laboratory of genetics and physiology 2; CARD, caspase activation and recruitment domains; CTD, c-terminal domain. A, acetylation; P, phosphorylation; U, ubiquitination; S, SUMOylation.

The infection of ECs with virus and other pathogens can trigger effective innate immune response in host cells to confine pathogen invasion. This process subsequently alerts neighboring immune cells to viral infections encountered. Pattern-recognition receptors (PRRs), including toll like receptors (TLRs), NOD-like receptors (NOD-like receptors, NLRs) and RIG-I like receptors (RLRs) (15), which specifically recognize the PAMP. The RLRs are a class of RNA helicases (containing DEX/DH box) that recognize virus-derived double strand RNA (dsRNA) and produce type I interferons (IFN-α and IFN-β) upon virus infection (16). RLRs family include three immune sensors, including retinoic acid-inducible gene I (RIG-I)/DDX58, melanoma differentiation-associated gene 5 (MDA5)/IFIH1, and laboratory of genetics and physiology 2 (LGP2)/DHX58 (17). Domain structural of RIG-I, MDA-5 and LGP2 was summarized in **Figure 2A** (18).

After activation by immunostimulatory viral RNA, RIG-I/MDA5 undergo conformational changes and CARD domain multimerization which allows RIG-I/MDA5 to interact with MAVS (mitochondrial antiviral signaling protein, also known as VISA), then transduces the signal to TRAF3 (TNF receptor associated factor 3), TBK1 (TANK binding kinase 1), IKKϵ (IκB kinase-ϵ) leading to increased phosphorylation, dimerization/oligomerization and nuclear translocation of IRF-3 (interferon regulatory factors-3) and IRF-7 as well as the activation of NF-κB and STATs (signal transducer and activator of transcription) (19). This process leads to increased expression of genes implicated in anti-viral response, such as type I interferons, interferon-stimulated genes (ISGs, such as ISG15 and ISG56), and pro-inflammatory factors, thereby delaying viral replication and transmission (18). RIG-I and MDA5 can recognize viral RNAs released from dengue virus, Nile virus, and reovirus. Uncapped 5’-triphosphate RNA (now termed 3pRNA) released from virus can directly be recognized by and binds to RIG-I (20). The activation of RIG-I can be dynamically regulated by several types of post-translational modifications. For example, TRIM25 (tripartite motif containing 25) promotes K63-linked ubiquitination in the CARD domain of RIG-I, leading to the activation of RIG-I downstream signaling activity (21). However, another E3 ligase RNF125, promotes K48-linked ubiquitination and degradation of RIG-I, thereby dampening anti-viral responses (22). Apart from multisite ubiquitination, other posttranslational modifications such as acetylation, SUMOylation and phosphorylation also play essential roles in regulating RIG-I function which has been recently reviewed elsewhere (18).

## Role of RIG-I in Regulating Endothelial Function

### Endothelium-Dependent Relaxation

Endothelial dysfunction is a constellation of cellular events consisting of impaired vascular relaxation, increased inflammation and leukocyte adhesion, endothelial cell senescence, endothelial mesenchymal transition etc (1). It is being recognized that inflammation and immune mechanisms are involved in endothelial activation and dysfunction *via* PRR. However, the impact of endothelial RIG-I activation on endothelial dysfunction is largely unexplored area. To address the role of RIG-I activation in endothelial function, Asdonk et al. (23) performed an experiment to inject RIG-ligand 3pRNA intravenously into mice and observed that RIG-I activation significantly impaired endothelium-dependent vasodilation in mouse aorta. In addition, RIG-I activation by 3pRNA leads to increased oxidative stress in aortic segments. Mechanistically, 3pRNA stimulation increased RIG-I expression in cultured human coronary ECs (HCAEC) as well as generation of reactive oxygen species (ROS), without affecting cell apoptosis or proliferation (23). In addition, RIG-I stimulation with 3pRNA results in increased expression of pro-inflammatory cytokines (such as IL-6 and IP-10). This study provides the first evidence showing activation of RIG-I by 3pRNA leads to endothelial activation and dysfunction. It remains unclear whether RIG-I can induce endothelial damage and atherogenesis *in vivo*. Further studies in endothelial cell conditional knockout mice will provide the answer to question whether endothelial cell derived nitric oxide (NO) production and bioavailability is impaired after RIG-I activation by dsRNA virus, nuclei acid ligands or its mimics.

### Endothelial Inflammation

Increased endothelial inflammation leads to endothelial dysfunction by recruiting monocytes, neutrophils and platelets to the inflamed surface area of vascular endothelium. It has been shown that bacterial lipopolysaccharides (LPS) can induce RIG-I expression dose-dependently (100 ng/ml to 10 μg/ml). Gain-of-function of RIG-I increased COX-2 (also known as PTGS2) gene expression by increasing COX-2 gene promoter (-1838 bp to +129 bp) activity (24). COX-2 is well recognized for its role in prostaglandin production and inflammation. It has been reported that selective inhibition of COX-2 activity improves endothelium-dependent vasodilation and reduces oxidative stress and inflammation in patients with coronary artery disease (25). Further studies are warranted to elucidate the detailed mechanisms that explain the upregulation of RIG-I by LPS and other inflammatory cytokines. It also remains elusive whether other inflammatory cytokines, such as TNF-α, IL-1β and IL-6 have similar RIG-I activating effects in ECs. Interventional studies in cultured cells and *in vivo* will elucidate decisive role of RIG-I in regulating endothelial inflammation.

### Endothelial Senescence

ECs will become senescent upon DNA damage, or exposure to multiple risk factors, such as smoking, sedentary lifestyle, oxidative stress and irradiation etc (26). Once become senescent, ECs will have impaired capacity to produce nitric oxide and cause vasoconstriction (27). In addition, senescent ECs will acquire senescence-associated secretory phenotype (SASP), which include the secretion of TNF-α, IL-1β, IL-6, IL-8, CCL2, PAI-1, MMP-2, MMP-9 and VEGF (28). The phenomenon of SASP will also impact neighboring cells *via* paracrine functions. Virus-induced senescence is commonly seen in many viral diseases (29). However, it remains unknown whether RIG-I play a role in SASP and whether there are therapeutic strategies that can combat senescence. To this end, Liu et al. (30) demonstrate that the expression of RIG-I gene and protein is induced, while that of klotho (an anti-aging molecule) was decreased in replicative senescent ECs. However, the expression of MDA5 (another member of RLR family) was not altered, suggesting the specific role of RIG-I in endothelial senescence. In contrast, depletion of RIG-I in senescent ECs decreases the secretion of IL-6 and IL-8. In addition, the authors observed that endogenous klotho interacts with RIG-I and inhibits IL-6 expression by blocking RIG-I multimerization mediated activation of NF-κB (30). This study implicates RIG-I as potential immune regulator of endothelial senescence and associated cardiovascular disorders.

### Angiogenesis, Endothelial Permeability and Nitric Oxide Generation

RIG-I also played an important role in angiogenesis, endothelial hyperpermeability and NO production. Poly (I:C), a potent activator of RIG-I signaling, has been shown to suppress VEGF-induced angiogenesis, vascular permeability *in vitro* and *in vivo (* 31). Notably, VEGF transgene-induced eNOS phosphorylation as well as upstream kinase Akt phosphorylation was attenuated by poly (I:C) treatment (31). The above consequences induced by poly (I:C) was abrogated by deletion of IFN receptor 1. These findings implicate that poly (I:C) treatment induced RIG-I activation have both Yin and Yang effects on endothelial function (31). The net effects of RIG-I activation on ECs need to be carefully examined in different disease context.

## Inducers of RIG-I Signaling in ECs

### Human Rhinovirus Infection

Human rhinovirus not only infects airway epithelium but also the vascular endothelium. It is reported that human rhinovirus infection leads to the activation of TLR3, RIG-I, and MDA5 and the expression of downstream genes, including IFN-β, RANTES, and IP-10 and OAS1, which was blocked by ICAM-1 blockade (32). This evidence suggests that human rhinovirus could infect ECs and instigate an anti-viral and inflammatory response, which can trigger leukocyte adhesion to ECs and endothelial activation (32).

### Hantaan Virus Infection

Hantaan virus (HTV) infection can cause hemorrhagic fever with renal syndrome which leads to high mortality (33). Upon HTV infection, RLR pathway is activated in human ECs, leading to activation of innate immunity, production of interferons, and expression of ISG (34). However, defense mechanisms against HTV infection remains largely unknown. A recent study has shown that circ_0000479 regulated RIG-I expression by sponging miR-149-5p, thereby delaying viral replication in HTV-infected human ECs. This study offers the first mechanism of epigenetic regulation of RIG-I expression in regulating HTV infection, thus offering new mechanistic insights into the precise mechanisms responsible for HTV-infection induced endothelial dysfunction (33).

### Porcine Circovirus Infection

Infection of ECs by porcine circovirus type 2 (PCV2) can cause endothelial dysfunction and vascular disorder associated with porcine circovirus disease (PCVD). When infected with PCV2, the expression of IL-8 was significantly increased in porcine iliac artery ECs (PIECs) *via* activation of RLR pathway, including RIG-I, MDA-5, MAVS and JNK signaling pathway, without affecting NF-κB signaling pathway. Meanwhile, the expression of endothelial-derived IL-8 was decreased by silencing RIG-I, MDA-5, or MAVS in PIECs or JNK inhibitor. These findings suggest that PCV2 infection can promote the activation of RIG-I/MDA-5/MAVS signaling axis to boost endothelial inflammation, providing novel mechanistic insights into the mechanism whereby PCV2 infection causes endothelial dysfunction and vascular failure (35).

### Dengue Virus

It has been well established that type I interferon or RIG-I agonists can mitigate the replication of Dengue virus (DENV) *via* RIG-I/MAVS/TBK1/IRF3-dependent antiviral responses (36). However, it remains unclear how DENV triggers endothelial cell activation and inflammation. Human brain microvascular ECs (HBMECs) are susceptible to DENV infection. After DENV infection, the expression of RIG-I is upregulated, followed by increased production of type I interferons and expression of various pro-inflammatory cytokines in a RIG-I dependent manner (37). In addition, after infection with DENV, the expression of pro-adhesive molecule ICAM-1 was increased in HBMECs in a RIG-I-dependent manner. These observations implicate that RIG-I activation by DENV promotes the release of adhesion molecules, which facilitates leukocyte recruitment to activated endothelium and propagate the vicious cycle of endothelial dysfunction (37).

### LPS

Lipopolysaccharide (LPS) derived from gram-negative bacteria, can activate ECs by secreting various pro-inflammatory cytokines/chemokines/adhesion molecules, such as ICAM-1, VCAM-1 and E-selectin (38). In 2002, Imaizumi et al. (24) provide the first experimental evidence showing that LPS can induce the expression of RIG-I and COX-2 in human ECs. Overexpression of RIG-I increases COX-2 promoter activity and gene expression (24). LPS-induced RIG-I activation has been subsequently verified by another study (39). Since IRF-1 functions downstream of LPS mediated pro-inflammatory pathways in ECs (24), RIG-I was identified as an important regulator of LPS-mediated IRF-1 induction and VCAM-1 upregulation (40). Further studies are warranted to elucidate the detailed mechanisms underlying LPS induced RIG-I activation and whether type I interferon pathway is involved in the effects of RIG-I in ECs.

### dsRNA

Poly (I:C) is a type of synthetic double-stranded RNA (dsRNA) that can enter ECs *via* clathrin endocytosis and trigger RIG-I activation in HUVECs (41) as well as glomerular ECs (42). Poly (I:C)-treated ECs produce inflammatory cytokines, such as IL-6, CCL2, CCL5, CXCL10, as well as type I interferons, such as IFN-α and IFN-β. Poly (I:C) induced pro-inflammatory effects is RIG-I dependent, as RIG-I siRNA, but not MDA5 siRNA abrogated poly (I:C)-induced endothelial inflammation and activation (42). Poly (I:C) treatment also increases the expression of adhesion molecules ICAM-1 and increased endothelial permeability. Therefore, poly (I:C) induced RIG-I upregulation activates type I interferon pathway and confer antiviral responses in ECs (42). This evidence provides molecular insights into the pathomechanisms by which viral infections trigger glomerulonephritis. Similarly, another study has demonstrated that treatment of porcine kidney ECs with poly (I:C) leads to the upregulation of ISG15 and ISG43 as well as TLR3, RIG-I and MDA5. However, mRNA expression of IFN-α and IFN-β was not affected, suggesting that poly (I:C) induced upregulation of ISG15 and ISG43 in ECs were mediated through IFN-independent pathway (43). Further studies are needed to clarify whether RIG-I mediated anti-viral response and the expression of ISGs will lead to endothelial activation in ECs from arterial vascular beds, such as the coronary artery.

### dsDNA

Double-stranded DNA (dsDNA) is a common type of PAMP that potently stimulates innate immunity after binding to PRR during sterile inflammation or viral infections. The role of dsDNA during infections has been well characterized in immune cells, however, dsDNA signaling and its pathophysiological consequences in ECs remain poorly defined. To understand the role of dsDNA in regulating endothelial function, Erik Gaitzsch et al. (44) transfected human ECs with poly(dA:dT) (a synthetic type of dsDNA) and observed that poly(dA:dT) treatment resulted in RIG-I activation in ECs. Poly(dA:dT) also increased expression of tissue factor (TF) and PAI-1 (plasminogen activator inhibitor-1), two well-established prothrombotic molecules in atherothrombosis, accelerated blood clot formation in a RIG-I-dependent manner (44). dsDNA treatment also leads to increased expression of vWF (von Willebrand Factor) and augments the interaction between platelet and activated endothelium under flow conditions (44). This evidence suggests that dsDNA released from DNA viruses can trigger a prothrombotic phenotype in the vascular endothelium of vascular beds, thus providing a novel insight into the interplay between innate immunity and thrombosis.

### IFN-γ

IFN-γ has been demonstrated to induce the upregulation of multiple pro-inflammatory genes in ECs, including ICAM-1 and VCAM-1 (45). IFN-γ also induces RIG-I expression and activation in human ECs (46). Interestingly, RIG-I gene upregulation induced by IFN-γ was not altered by the treatment with cycloheximide, a pharmacological inhibitor of protein translation (46). RIG-I protein was also expressed in normal lung endothelium (46). Although the biological function of RIG-I in ECs is largely unknown, RIG-I induction by IFN-γ indicate that RIG-I could be important in inflammatory or immune disorders.

### 25-Hydroxycholesterol (25-HC)

25-hydroxycholesterol (25-HC) is an oxysterol involved in vascular inflammation and interference with viral entry and replication (47). It has been reported that RIG-I expression can be increased by treatment with 25-HC. Mechanistic studies revealed that 25-HC induces RIG-I expression and downstream genes including IL-8 in an IRF1-dependent manner (48). RIG-I transduces the signal of 25-HC by binding to its downstream molecules-MAVS, TBK1, and MAPK, which caused the activation of transcription factors, such as NF-κB and AP-1 (activator protein-1). More importantly, RIG-I protein is highly expressed in atherosclerotic lesions (48). These evidences support the concept that RIG-I signaling is involved in vascular inflammation and atherosclerotic plaque formation. Targeting RIG-I and its downstream pro-inflammatory signaling offers a new potential therapy for atherosclerosis.

### Angiotensin II (Ang-II)

Angiotensin II (Ang-II) is a potent stimulus for the proliferation and migration of vascular smooth muscle cells and ECs, leading to the development of hypertension and vascular remodeling (49). The proliferation, migration of lymphatic ECs (LECs) and tube-like formation are key cellular events for lymphangiogenesis under patho-physiological conditions (50). Ang-II treatment markedly induced lymphangiogenesis in cultured LECs and in mouse hearts by increased expression of genes in the RLR pathway including RIG-I. The effect of Ang-II on RIG-I expression and lymphangiogenesis was reversed by treatment with losartan, a pharmacological inhibitor of Ang-II type 1 receptor (AT1R) (50). These findings indicate that Ang-II can regulate lymphangiogenesis in LECs *via* AT1R, highlighting the potential of AT1R blockers to treat hypertension and associated cardiovascular remodeling by targeting RIG-I.

## Pharmacological Modifiers of RIG-I Signaling in ECs

### Panax Notoginseng Saponins (PNS)

Based on above literature, mounting evidence suggests that RIG-I activation is strongly associated with endothelial activation and dysfunction. Strategies that block RIG-I activation have the potential to reduces endothelial dysfunction associated with RIG-I activation caused by viral infection, LPS or other disease-relevant stimuli. *Panax notoginseng* saponins (PNS) is the main pharmacologically active constituent of a *Panax notoginseng*, an eminent traditional Chinese medicinal herb that has been used in China for treating cardiovascular diseases for many years (51). However, the underlying mechanism-of-action of PNS in preventing endothelial dysfunction remain elusive. To this end, Zhang et al. (52) evaluated the effects and mechanism of PNS in treating cerebral ischemia by utilizing the cellular model of oxygen-glucose-deprivation (OGD) in cultured brain microvascular ECs (BMECs). Microarray analysis was performed to profile differentially expressed genes in BMECs exposed to PNS treatment. It was found that PNS suppresses the RLR and NF-κB pathways (52). In rats undergoing middle cerebral artery occlusion (MCAO), PNS also decreases the levels of the downstream cytokines (TNF-α and IL-8) *via* suppressing RLR pathway, followed by neurological improvement and reduced infiltration of inflammatory cells in brain tissues (52). These evidences provide novel mechanistic insights into the endothelial protective effects of PNS against cerebral ischemia *via* suppressing RIG-I pathway (52).

### 15d-PGJ2

Emerging evidences have shown that RIG-I is expressed in ECs that sense DAMP and PAMP. To identify potential pharmacological regulators of RIG-I in ECs, Imaizumi et al. (39) explored the potential role of PPAR-γ in LPS-induced RIG-I expression in HUVECs by focusing on15d-PGJ2, an endogenous PPAR-γ ligand that exerts anti-inflammatory effects. In addition, it has also been reported that 15d-PGJ2 can block TNF-α-induced adhesion of monocytes to ECs by decreasing the expression of adhesion molecules *via* inhibiting NF-κB pathway (53) as well as covalent modification of the proteasome components (54). Besides these mechanisms, 15d-PGJ2, but not ciglitazone, attenuates LPS (1 μg/ml) -induced RIG-I expression in HUVECs in time-and dose-dependent manners (39). These findings implicate that 15d-PGJ2 attenuates LPS-induced RIG-I upregulation through a PPAR-γ-independent mechanism (39). Together, these evidences indicate that RIG-I inhibition could at least partially mediate the inhibitory effects of 15d-PGJ2 on the adhesion and infiltration of leukocytes across the brain endothelium, underlying its pleiotropic effects in cardiovascular and inflammatory disorders.

### Tryptanthrin

Tryptanthrin is a bioactive constituent isolated from indigo plants such as *Polygonum tinctrorium*. Tryptanthrin has well-established anti-inflammatory effects in various disease contexts (55, 56). To evaluate the pharmacological effects of tryptanthrin on the production of pro-inflammatory cytokine/chemokine in HUVECs, Kawaguchi et al. (57) found that tryptanthrin suppressed RIG-I activation and the expression of RIG-I downstream genes including IFN-β, CXCL10, ISG15 and IFITM1 in HUVECs exposed to poly (I:C), a TLR3 ligand, without affecting poly IC-induced activation of IRF3 (57). Mechanistic studies revealed that tryptanthrin reduced the nuclear translocation of STAT1 in HUVECs exposed to poly (I:C). These findings implicate that tryptanthrin inhibited poly (I:C)-induced upregulated expression of ISGs *via* blocking STAT1 activation in HUVECs, establishing tryptanthrin as a promising therapeutic agent to halt TLR3-mediated vascular inflammation *via* suppressing RIG-I activation (57).

### 2-Aminopurine

Viral infection can trigger immune responses in ECs. To mimic viral infection of host cells including ECs, poly (I:C) is a widely used stimulus. Poly (I:C) treatment of cells leads to the phosphorylation of protein kinase R (PKR), thereby contributing to the induction of type I interferons-mediated antiviral responses. 2-Aminopurine (2-AP) is a potent inhibitor of dsRNA-activated PKR. Treatment of HUVECs with poly (I:C) induced the expression of RIG-I. However, 2-AP attenuates poly (I:C)-induced RIG-I upregulation. To correlate this finding *in vivo*, hantavirus infection also leads to RIG-I upregulation in rat endothelium by immunohistochemical staining (41). In addition, 2-AP could also significantly attenuated palmitate-induced endothelial senescence by preventing PKR-dependent JNK activation and SIRT1 downregulation (58). These findings indicates that RIG-I could be involved in antiviral responses as well as pro-senescent effects in ECs exposed to viral infection and free fatty acid. These findings also suggest that 2-AP could be a pharmacological inhibitor of the anti-viral as well as pro-senescent response of RIG-I.

## Concluding Remarks and Future Perspective

In summary, ECs play a vital role in innate immunity against bacterial and viral infections by sensing PAMPs and DAMPs as well as producing a wide range of innate immunity proteins (13). These endothelial cell-derived proteins limit bacterial and viral infections *via* diverse mechanisms, including type I interferon anti-viral response (13). RIG-I activation in ECs could be a double-edged sword in health and diseases. On the one hand, endothelial RIG-I activation upon viral infection could confer anti-viral responses; on the other hand, virus infection can also trigger vascular inflammation, endothelial dysfunction, coagulation and cardiovascular diseases.

Similarly, bacterial-infection will trigger a host response, contributing to both killing/clearance of pathogenic bacteria as well as causing tissue/organ damage (59). For example, rickettsia (a causative agent of Mediterranean spotted fever), can also infect vascular endothelium, and augments the expression of pro-inflammatory cytokines/chemokines (IL-6 and L-8), redox-sensitive genes (HMOX-1), IFN-β, tissue factor, vasoactive prostaglandins, ISG and platelet/leukocyte adhesion/transmigration and coagulation process *via* activation of RIG-I and NF-kB (59–66). The eventual consequence is leukocyte infiltration and tissue damage. The second example is the infection of fibroblasts with Chlamydia pneumoniae which induced the expression of interferon-stimulated genes dependent on TLRs and RIG-I, albeit Chlamydia pneumoniae-induced type 1 IFN response exhibit a delayed kinetic profile, compared with the response occurred during virus infections (67).

Caution should be taken when thinking to activate RIG-I signaling pharmacologically or immunologically in ECs. It has been reported that immune activation of ECs inhibits HIV replication in macrophages (68). The specific consequence of RIG-I activation in ECs need to be carefully analyzed systematically, including the role of RIG-I in virus- and bacteria-induced endothelial senescence, oxidative stress, EndoMT, and coagulation. Since viral pathogens in particular are novel activators of inflammatory responses and procoagulant signalling (69), all of these will trigger the development of atherosclerotic cardiovascular disease.

RLR pathway cooperates and crosstalk with other PRR, such as TLRs, cGAS-STING, NLRP3 inflammasome to regulate adaptive immune response and inflammation (17, 70). In light of pro-atherogenic effects elicited by activation of TLRs, cGAS-STING, NLRP3 inflammasome pathways, the precise role of endothelial RIG-I activation in atherosclerosis remains to be validated in RIG-I conditional knockout mice to dissect the specific role of RIG-I of vascular cells in atherosclerosis.

Also, given the important role of trained immunity in regulating endothelial function (70–72), further studies are warranted to reveal whether virus, bacteria or disease-relevant stimuli induced RIG-I activation will elicit long-lasting effects in ECs. Furthermore, recent evidence has suggested that SARS-CoV2 infection leads to multiple aspects of endothelial dysfunction, ranging from leukocyte adhesion, virus-induced senescence, EndoMT etc (1). However, it remains unknown whether RIG-I play a significant role in COVID-19-associated endothelial dysfunction and whether SARS-CoV-2 infection can dampen anti-viral responses in the ECs considering the fact that this virus destroys the RLR signaling pathway to escape the cellular immune response (73).

Lastly, the use of biotechnological advances such as quantitative proteomic analysis of the endothelial secretome (74) and single cell RNA-sequencing of infected blood vessels will elucidate differential proteins/targets in ECs subject to bacterial and viral infection.

In conclusion, the specific role of RIG-I activation on endothelial function is an important research direction to further our understanding in the area. The regulation of RIG-I by multiple infection caused by virus and bacteria, atherosclerosis-relevant stimuli and pharmacological modifiers suggest that RIG-I might be therapeutically targetable (**Figure 2B**). Picturing the role of RIG-I activation in early and late stages of viral infection is pivotal to understand how vascular ECs are involved in immunity and the modulation of the host response. Further translational studies on elucidating the role of RIG-I signaling in ECs may identify new therapeutic targets for the treatment of cardiometabolic and infectious disease arising from bacterial and viral infections.

## Author Contributions

Conceptualization: SX and JW. Writing: SX. Revision: SX, TJ, and JW. All authors contributed to the article and approved the submitted version.

## Funding

This study was supported by grants from National Key R&D Program of China (No.2021YFC2500500), National Natural Science Foundation of China (Grant Nos. 82070464, 81941022, 81530025) and Strategic Priority Research Program of Chinese Academy of Sciences (Grant No. XDB38010100). This work was also supported by Program for Innovative Research Team of The First Affiliated Hospital of USTC (CXGG02), Anhui Provincial Key Research and Development Program (Grant No. 202104j07020051), Local Innovative and Research Teams Project of Guangdong Pearl River Talents Program (Grant No. 2017BT01S131).

## Conflict of Interest

The authors declare that the research was conducted in the absence of any commercial or financial relationships that could be construed as a potential conflict of interest.

## Publisher’s Note

All claims expressed in this article are solely those of the authors and do not necessarily represent those of their affiliated organizations, or those of the publisher, the editors and the reviewers. Any product that may be evaluated in this article, or claim that may be made by its manufacturer, is not guaranteed or endorsed by the publisher.
